# An Automated Sequential Computational Screening Workflow for Hydrogen Diffusion, Structural Response, and Fatigue Analysis of EPDM O-Rings Under High-Pressure Hydrogen Exposure

**DOI:** 10.3390/polym18172115

**Published:** 2026-08-31

**Authors:** Hanmin Park, Hansang Kim

**Affiliations:** Department of Mechanical Engineering, Gachon University, Seongnam 13120, Republic of Korea; hanmin610@gachon.ac.kr

**Keywords:** EPDM, hydrogen, O-ring, mass diffusion, swelling, hyperelasticity, fatigue crack growth, tearing energy, computational screening

## Abstract

Hydrogen transport and cyclic loading complicate O-ring assessment. This study developed an automated sequential screening workflow for ethylene–propylene–diene monomer (EPDM) O-rings, linking Abaqus mass diffusion, one-way concentration-field transfer to nonlinear structural/contact analysis with prescribed isotropic swelling, and fe-safe/Rubber fatigue post-processing. EPDM 60A and 90A were evaluated at sampled pressures of 3, 7, 14, and 21 MPa and circumferential installation stretches of 1–5%, with radial squeeze fixed at 20%. Structural analyses used third-order Reduced Polynomial models with built-in Mullins effects. Material-specific diffusivities characterized at approximately 1 bar were held constant across pressures; hydrogen-content and post-decompression free-specimen volume-change data informed model inputs. Fatigue inputs were characterized in air at approximately 23 °C using non-equivalent bases for the two compounds. At 1% stretch, fatigue-life outputs decreased from 6.877 × 10^7^ to 12.923 repeats for 60A and from 3.155 × 10^9^ to 3.896 repeats for 90A between 7 and 14 MPa. At 21 MPa, outputs were 0.712 and 0.041 repeats, respectively; these fractional values are numerical screening indicators without assignment to a within-cycle event or damage mode. The outputs support only input-conditional comparison of axisymmetric cases and experimental prioritization; they do not support decisions on component durability, leakage, sealing performance, or operating pressure.

## 1. Introduction

Effects of high-pressure hydrogen exposure on filled EPDM sealing composites have been reported [1]. Reliable sealing at joints, valves, and connectors is therefore an important engineering requirement for hydrogen infrastructure [2,3]. These considerations motivate analysis of the nominal response of installed O-rings while maintaining a clear distinction between computational screening and component-level validation.

Hydrogen exposure of elastomeric seals can be associated with several distinct phenomena, including molecular transport, macroscopic volume change, rapid gas decompression (RGD) damage, blister formation, compression-set evolution, and fatigue-related damage [3,4,5,6,7]. These processes should not be treated as interchangeable. Hydrogen transport and macroscopic volume change concern mass uptake and bulk deformation [6,7], whereas RGD and blistering involve localized cavity and fracture processes [3,4,5,6]. The present study is limited to an effective diffusion field, an empirically prescribed swelling strain, nonlinear structural/contact response, and downstream small-crack fatigue post-processing. It does not explicitly simulate cavity nucleation, blister growth, an evolving geometric crack, or hydrogen-dependent changes in the hyperelastic constitutive parameters.

Component-level durability testing under cyclic high-pressure hydrogen can be experimentally demanding because of specialized facilities, test duration, safety requirements, and the number of material and assembly variables [3,4]. Computational analysis can therefore complement, but not replace, such testing by screening predefined nominal cases and identifying conditions that warrant further investigation. Prior studies have addressed high-pressure-hydrogen O-ring damage and transport [3,6], swelling [7], sealing-life assessment [8], and multiaxial elastomer fatigue [9] as distinct topics. The present work coordinates selected transport, swelling, structural/contact, and fatigue-post-processing stages within one automated sequence. Four sampled pressure points—3, 7, 14, and 21 MPa—are evaluated; they do not define a continuous pressure threshold, allowable pressure, or safety factor.

For O-ring applications, installation geometry, squeeze, contact, constitutive nonlinearity, and cyclic pressure loading can influence the local structural history subsequently evaluated for fatigue. A nominal axisymmetric representation cannot capture all three-dimensional assembly tolerances, local defects, surface roughness, or asymmetric extrusion features that may occur in service. The present workflow therefore distinguishes the nominal modeled response from component-level durability and leakage performance. Radial squeeze is fixed at 20%, circumferential installation stretch is varied from 1% to 5%, and no extrusion clearance gap or explicit leakage model is included.

Accordingly, this study develops an automated sequential diffusion–structural–fatigue screening workflow for EPDM 60A and 90A O-rings under selected high-pressure hydrogen exposure conditions. Abaqus is first used to calculate the effective hydrogen concentration field, which is transferred one way to a nonlinear structural/contact analysis with a prescribed concentration-dependent swelling input. The Cycle-2 structural history is then passed downstream to fe-safe/Rubber for tearing-energy-based fatigue post-processing [9,10]. Isight executes the predefined material–pressure–stretch design matrix and routes the corresponding inputs and outputs [11]. The study reports deterministic structural and fatigue outputs at 3, 7, 14, and 21 MPa and 1–5% initial circumferential stretch with radial squeeze fixed at 20%, examines pressure- and stretch-dependent numerical trends within the nominal axisymmetric configuration, and identifies limitations associated with the transport, swelling, fatigue, and geometry assumptions. The intended contribution is a transparent screening workflow that can help prioritize subsequent experiments and higher-fidelity analyses; it does not replace component-level validation or provide service-life, leakage, or allowable-pressure predictions.

## 2. Theoretical Background and Literature Review

The present study represents the response of rubber seals under high-pressure hydrogen exposure through sequential computational stages rather than a bidirectionally coupled solution. The workflow comprises an effective Fickian transport analysis, a prescribed concentration-dependent swelling strain, nonlinear hyperelastic structural analysis, and downstream tearing-energy-based fatigue post-processing.

### 2.1. Hydrogen Mass Diffusion and Permeation Mechanisms

The movement of hydrogen molecules within the rubber matrix involves sorption at the interface and diffusion through the free volume inside the polymer. During permeation, hydrogen molecules enter the polymer and progressively occupy the available free volume; upon depressurization, they re-emit to restore equilibrium with the external environment [12,13]. Under typical low- to medium-pressure conditions, the equilibrium concentration of dissolved hydrogen, $C_{\infty }$, follows Henry’s law and is proportional to the external pressure p:

(1) $$C_{\infty }=k_{h}\cdot p$$

Hydrogen uptake in elastomers may involve multiple physical contributions, including dissolution in the polymer matrix and interactions with microstructural features or fillers [14,15,16]. The present model does not resolve these contributions separately. Instead, it represents hydrogen transport using one effective concentration field and distinguishes the physical transport solubility measured under the approximately 1-bar characterization condition from the empirical pressure-dependent wt.ppm scale used in the high-pressure analyses.

Fick’s law of diffusion describes the spatiotemporal evolution of the hydrogen concentration field C(x,t):

(2) $$\frac{\partial C}{\partial t}=\nabla \cdot \left(D\nabla C\right)$$

where *D*_H_ represents the material-specific effective diffusivity. The resulting concentration field is used in the present workflow only as a transport quantity that is transferred one way to the structural model; cavity evolution, crack initiation, and crack growth are not explicitly solved in the diffusion analysis.

### 2.2. Computational Implementation of Mass Diffusion

For the implemented high-pressure analyses, the modeled hydrogen concentration was normalized using the empirical pressure-dependent hydrogen-content scale, *C*_scale_(*P*), on the same wt.ppm basis:

(3) $$\phi \left(x,t;P\right)=\frac{C_{H}\left(x,t;P\right)}{C_{scale}\left(P\right)}$$

Here, *C*_H_ denotes the effective modeled hydrogen concentration, *C*_scale_(*P*) denotes the pressure-dependent empirical input scale described in Section 3.2, and *ϕ* is dimensionless. Both *C*_H_ and *C*_scale_(*P*) were represented on the same wt.ppm basis. These pressure-point values were used directly as the Abaqus solubility input, and no additional molar or SI concentration conversion was applied to this normalization. The physical transport solubility measured under the approximately 1-bar condition is denoted separately by *S*_H_. The effective Fickian equation given above was used with a material-specific diffusivity *D*_H_. Thermal-gradient and hydrostatic-stress-driven contributions available in Abaqus were omitted [17]. Structural stress and deformation did not feed back to the transport solution; the transport-to-structure transfer was therefore one-way. In this notation, *P* denotes the discrete applied-pressure input for each simulated case (3, 7, 14, or 21 MPa), whereas *p* in Equation (1) denotes the general external pressure in Henry’s law.

In the implemented charging calculation, the hydrogen-exposed boundary was assigned the model normalization state *ϕ* = 1. This boundary condition is a computational normalization and is not interpreted here as independent proof of a species-resolved physical saturation state.

### 2.3. Hydrogen-Induced Swelling

Hydrogen exposure can be associated with changes in elastomer volume. In this study, swelling was represented empirically using post-decompression free-specimen volume measurements; in-situ swelling under pressure was not measured.

The measured post-decompression free-specimen volume-change ratio was first converted algebraically to a free-specimen equivalent isotropic strain [18]:

(4) $$\varepsilon_{free,eq}\left(P\right)={\left(1+\frac{\Delta V}{V_{0}}\right)}^{1/3}-1$$

(5) $$\varepsilon_{sw}\left(x,t;P\right)=\alpha_{sw,eff}\left(P\right)\;\phi \left(x,t;P\right)I$$

The structural model used the second relation as an effective pressure-dependent isotropic swelling-strain input scaled by the normalized concentration field. The dimensionless quantity *α*_sw,eff_ is the swelling-strain input at full normalization, *ϕ* = 1. The measured volume change, the derived free-specimen equivalent strain, and the prescribed structural input are distinct quantities. The transfer from a free specimen to a groove-constrained O-ring is an empirical modeling assumption and was not independently validated.

### 2.4. Hyperelasticity and the Mullins Effect

The reported O-ring structural analyses used the Abaqus third-order Reduced Polynomial hyperelastic model (*N* = 3) for both EPDM compounds. The Abaqus Reduced Polynomial form with *N* = 3 is Yeoh-equivalent; the term “Reduced Polynomial” is used here because it corresponds to the implemented material definition. The undamaged strain-energy potential is written as:

(6) $$U_{0}=\sum_{i=1}^{3}C_{i0}{\left({\bar{I}}_{1}-3\right)}^{i}+\sum_{i\in A}\frac{1}{D_{i}^{RP}}{\left(J_{el}-1\right)}^{2i}$$

Here, ${\bar{I}}_{1}$ is the first invariant of the deviatoric strain tensor, *J*_el_ is the elastic volume ratio, and *A* denotes the active volumetric terms. For EPDM 60A, only the $D_{1}^{\mathrm{RP}}$ term was active and $D_{2}^{\mathrm{RP}}=D_{3}^{\mathrm{RP}}=0$ was recorded. For EPDM 90A, the nonzero $D_{1}^{\mathrm{RP}}$, $D_{2}^{\mathrm{RP}}$, and $D_{3}^{\mathrm{RP}}$ inputs described in Section 3.1 were retained. An Ogden calibration existed as a non-controlling alternative but did not generate the reported O-ring results and is therefore not included in the reported constitutive input set. Mechanical stress softening was represented using the Abaqus built-in Mullins effect; no user-defined UMULLINS routine was used. In the built-in pseudo-elastic formulation, the energy and history variables are represented by:

(7) $$U=\eta {\tilde{U}}_{dev}+\Phi_{M}\left(\eta \right)+{\tilde{U}}_{vol}$$

(8) $$\eta =1-\frac{1}{r}erf\;\left[\frac{{\tilde{U}}_{dev}^{max}-{\tilde{U}}_{dev}}{m+\beta {\tilde{U}}_{dev}^{max}}\right]$$

Here, ${\tilde{U}}_{dev}^{max}$ is the maximum undamaged deviatoric strain-energy density previously experienced by the material point and ${\tilde{U}}_{dev}$ is its current value. The material-specific parameters *r*, *m*, and *β* correspond to the Reduced-Polynomial material branch described in Section 3.1. The parameters *r* and *β* are dimensionless, whereas *m* has energy-density units and is reported as MPa. These parameters were calibrated from non-hydrogen-conditioned mechanical data and were held fixed with hydrogen pressure and concentration. Accordingly, the Mullins model represents mechanical stress softening and is not a hydrogen-dependent degradation law.

### 2.5. Fracture Mechanics and Elastomeric Fatigue Life Prediction

Fatigue post-processing was performed using the fe-safe/Rubber tearing-energy-based small-crack formulation [9,19]. The method integrates an effective crack-growth life from an effective precursor size *c*_0_ to a configured integration endpoint c_f_. It does not insert an explicit crack into the Abaqus structural mesh and does not simulate geometric crack-path evolution, blister formation, or structural rupture.

Within the small-crack formulation, fe-safe/Rubber represents tearing energy as the product of a cracking-energy-density measure and effective crack size:

(9) $$T=K\;W_{c}\;c$$

where *K* is a dimensionless proportionality constant, *W*_c_ is the cracking-energy-density measure, and *c* is the effective crack size.

Under fully relaxing conditions, the material definitions used the fitted Thomas power-law fatigue-crack-growth relation:

(10) $$\frac{dc}{dN}=r_{c}{\left(\frac{T_{max}}{T_{c}}\right)}^{F_{0}}$$

Here, *T*_c_ is treated as a material parameter in the fitted crack-growth law and is not interpreted as a pressure threshold or a component-rupture criterion.

For EPDM 60A, compound-specific non-relaxing characterization was available, and the material definition used the Mars–Fatemi relations [9,10]:

(11) $$F\left(R\right)=F_{0}exp\left(F_{exp}R\right)$$

(12) $$T_{eq}=T_{max}^{F\left(R\right)/F_{0}}\;T_{c}^{1-F\left(R\right)/F_{0}}$$

These relations account for the load-ratio dependence of the fitted power-law behavior; no single representative *R* is assigned to the non-relaxing characterization as a whole. For EPDM 90A, only fully relaxing *R* = 0 fatigue-crack-growth characterization was available. Its fe-safe/Rubber material definition used THOMAS + NOCRYSTALLIZATION, with the configured noncrystallizing treatment expressed as follows:

(13) $$T_{eq}=\Delta T=T_{max}\left(1-R\right)$$

NOCRYSTALLIZATION is a solver/material-definition setting and is not experimental proof that EPDM 90A physically lacks strain crystallization. The two fatigue definitions are therefore methodologically non-equivalent, and the direction and magnitude of the bias associated with the 90A treatment are unknown. Finally, the numerical fatigue-life output is obtained by integrating the configured crack-growth law from the effective initial precursor size *c*_0_ to the configured integration endpoint *c*_f_:

(14) $$N=\int_{c_{0}}^{c_{f}}{\left(\frac{dc}{dN}\right)}^{-1}dc$$

The value *c*_f_ is a configured numerical integration endpoint and is not interpreted as a validated physical rupture size.

## 3. Material Characterization and Experimental Procedures

Two commercial EPDM compounds manufactured and supplied by Pyeong Hwa Oil Seal and identified by nominal Shore A hardnesses of 60 and 90 were investigated. Detailed compound identifiers, batch/lot information, filler formulation, cure system, and crosslink density were confidential or otherwise unavailable, and equivalence of the material batches used across the different characterization campaigns was not independently verified. These uncharacterized formulation and batch variables are treated as uncontrolled compound-specific differences and are not used as demonstrated causal explanations for the measured or numerical responses. The two materials were characterized and analyzed within the same nominal pressure–stretch matrix, while their fatigue-characterization bases remained non-equivalent as described in Section 3.3.

### 3.1. Hyperelastic Property Evaluation

To simulate the large-deformation response, Axel Products conducted simple tension, planar tension, and equi-biaxial tension tests under strain control. Repeated loading–unloading cycles were used to characterize mechanical stress softening. Based on the measured large-deformation data, the reported O-ring analyses used the third-order Reduced Polynomial model (*N* = 3) together with the corresponding material-specific Abaqus built-in Mullins-effect parameters. Table 1 reports only the constitutive inputs that generated the reported O-ring structural histories. An Ogden calibration was also generated during material evaluation but was not used in the reported O-ring analyses and is not included in Table 1.

### 3.2. Hydrogen Sorption and Swelling Characterization

The Korea Research Institute of Standards and Science (KRISS) characterized permeability, diffusivity, and physical transport solubility using a manometric method under an approximately 1-bar hydrogen condition [20]. These measurements provide the material-specific transport properties summarized in Table 2. In the computational analyses, one material-specific diffusivity was held constant across the 3, 7, 14, and 21 MPa cases; this pressure transfer is a modeling assumption rather than a demonstrated pressure-independent material property.

High-pressure exposure tests were separately used to obtain the post-decompression free-specimen volume-change observations and the pressure-dependent hydrogen-content values at four pressure points used as empirical model inputs [14,21].

**Figure 1:**
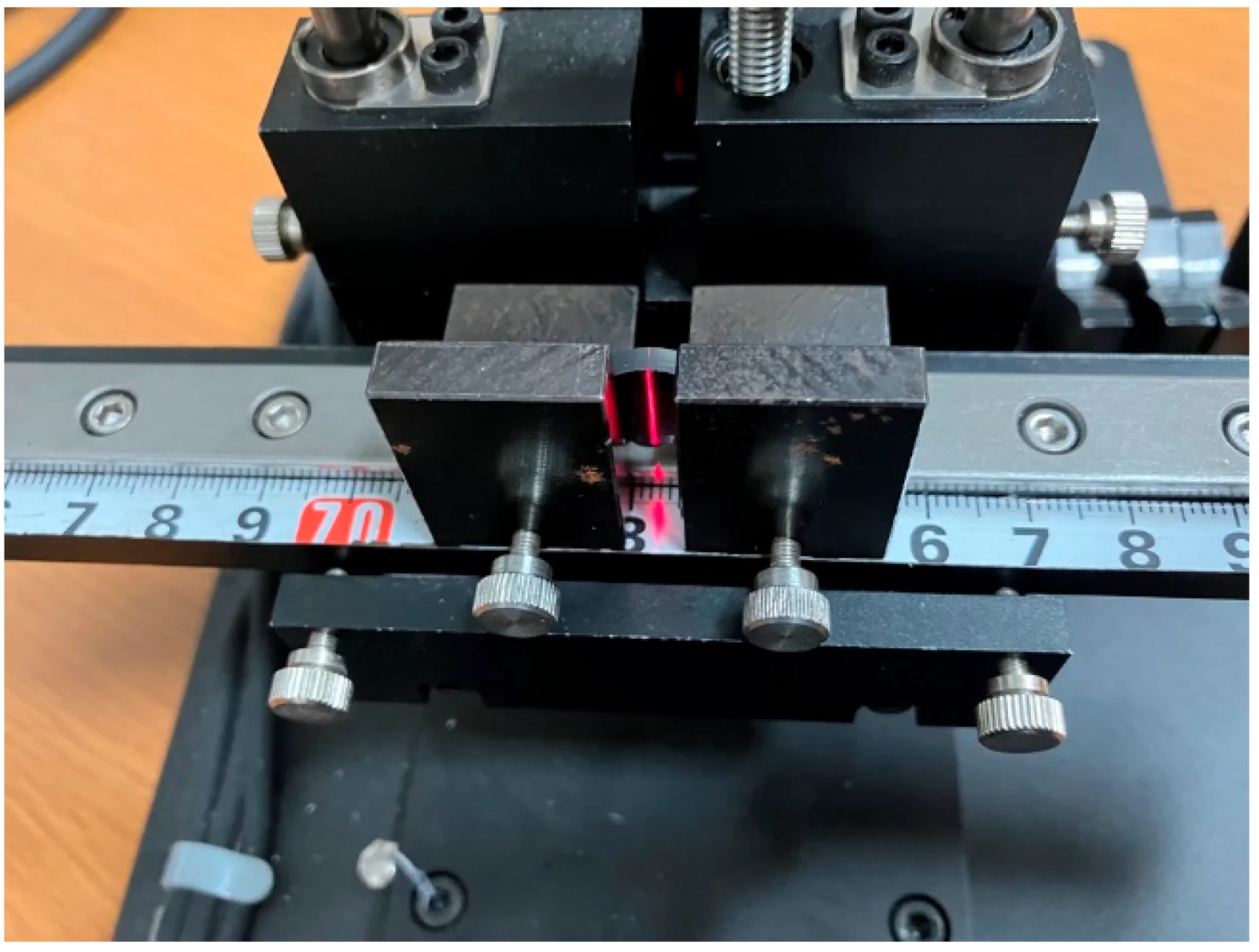
Experimental setup for the evaluation of hydrogen-induced volumetric swelling in EPDM specimens using a high-precision laser micrometer.

Disk-shaped specimens (12 mm in diameter and 2 mm in thickness for volume measurement; 15 mm in diameter and 2 mm in thickness for hydrogen-content measurement) were exposed to 99.9% hydrogen at 3, 7, 14, and 21 MPa and 25 °C for approximately 24 h. After rapid decompression, the free-specimen volume was measured immediately using the laser-micrometer setup shown in Figure 1. The reported volume quantity is therefore a post-decompression free-specimen volume-change ratio and is not treated as a direct measurement of the constrained O-ring swelling state.

The reported post-decompression volume changes at 3 and 7 MPa were small and included both positive and negative values. Because the available test records did not specify the number of replicates or whether the values represented individual measurements or averages, these results were not interpreted as statistically resolved zero- or nonzero-swelling responses. The reported values are retained in Table 3. In the structural model, the prescribed swelling input was set to zero at 3 and 7 MPa as a modeling simplification, while positive effective swelling-strain inputs were prescribed at 14 and 21 MPa. The zero-input choice is not an experimentally demonstrated zero-swelling condition.

The measured values, algebraically derived free-specimen equivalent strains, and prescribed structural swelling inputs are reported separately in Table 3.

**Figure 2:**
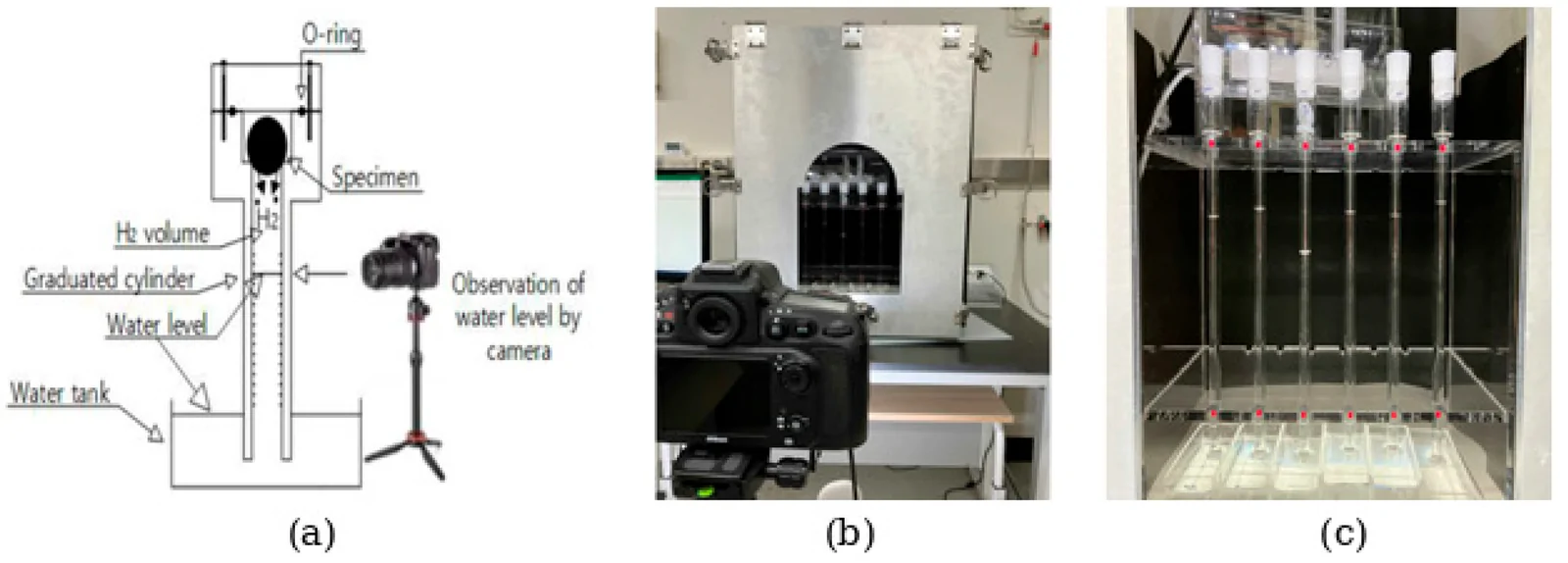
Experimental setup for measuring the volume of desorbed hydrogen gas using the water displacement method: (**a**) schematic diagram, (**b**) photographic views of the measurement system and (**c**) close-up view of the graduated-cylinder array in the water tank.

For the hydrogen content test, the residual hydrogen amount was calculated based on the principle that the release of hydrogen gas dissolved within the EPDM alters the water level in a sealed submerged cylinder [21], as shown in Figure 2.

Following exposure and rapid decompression, the residual hydrogen content was measured using the volumetric analysis device shown in Figure 2 and reported on a wt.ppm basis. Table 4 lists the measured hydrogen-content values at four pressure points associated with high-pressure exposure and decompression. These values were used directly as the pressure-dependent Abaqus solubility input and are denoted here as the empirical scale *C*_scale_(*P*). The modeled concentration *C*_H_ and *C*_scale_(*P*) were both treated on the wt.ppm basis, so that *ϕ* = *C*_H_/*C*_scale_(*P*); no additional unit conversion was applied. These pressure-point values do not resolve dissolved, adsorbed, trapped, or cavity-associated hydrogen populations separately.

The measured hydrogen contents differed between the two compounds and varied with pressure. Because detailed formulations and batch equivalence were not established, the present data do not isolate a unique effect of hardness, filler composition, crosslink density, or any other formulation variable. These values are therefore used as compound-specific empirical inputs rather than as evidence for a single composition-controlled mechanism.

### 3.3. Fatigue Crack Growth Characterization

Fatigue-related material properties used in this study were characterized in air at approximately 23 °C. Fully relaxing *R* = 0 fatigue-crack-growth data were available for both compounds. EPDM 60A was additionally characterized under non-relaxing *R* > 0 loading histories spanning multiple loading conditions; no single representative *R* is assigned to that dataset. The EPDM 60A material definition used the Mars–Fatemi non-relaxing treatment. No corresponding compound-specific non-relaxing fatigue-crack-growth dataset was available for EPDM 90A; its material definition therefore used THOMAS with the software setting NOCRYSTALLIZATION. The two fatigue definitions are not methodologically equivalent, and the bias associated with applying the 90A *R* = 0-based characterization to the O-ring history is unknown.

**Figure 3:**
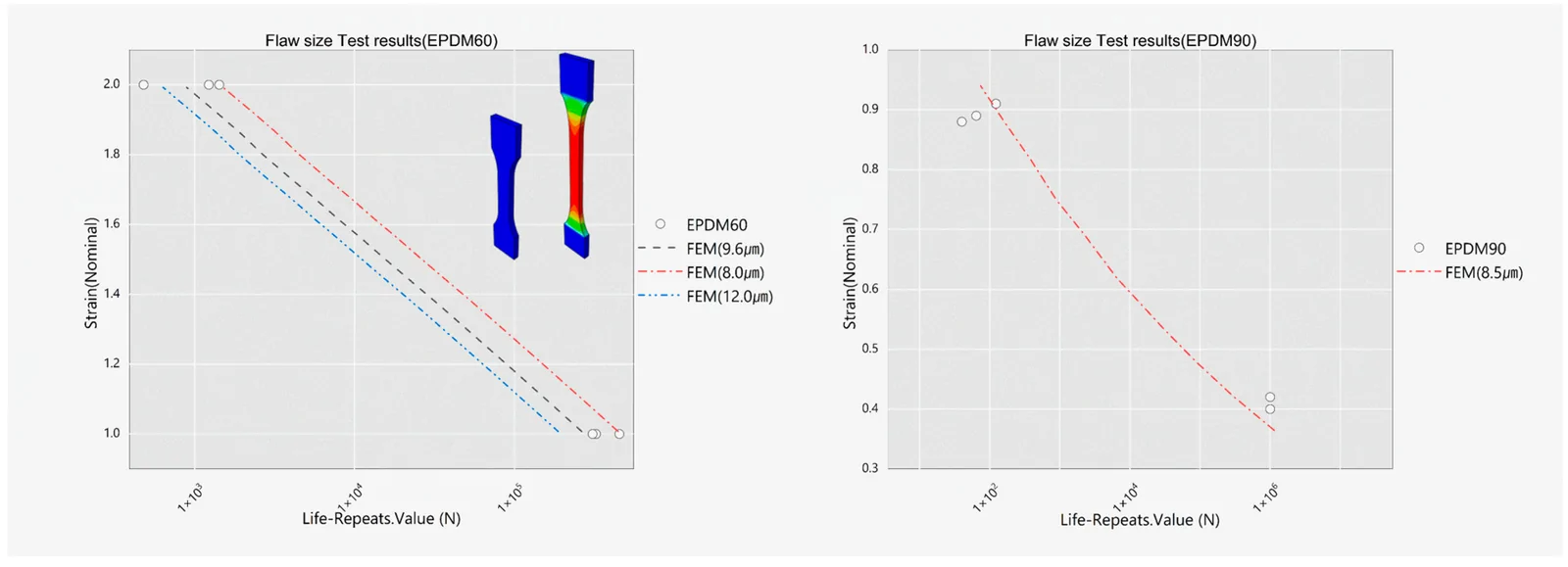
Available comparisons between unnotched fatigue-test observations and FEM life outputs used in the inverse calibration of the effective precursor-size inputs. The selected values were *c*_0_ = 0.0096 mm for EPDM 60A and *c*_0_ = 0.0085 mm for EPDM 90A; neither value is interpreted as a measured physical crack-nucleation size in the O-ring. The embedded FEM images show the undeformed and deformed model geometries.

1. Static Tearing Test: A planar tension specimen featuring a 25 mm notch was stretched under monotonic displacement until ultimate failure. The critical tearing energy ($T_{c}$) was calculated by integrating the strain energy density up to the point of failure. EPDM 60A exhibited a high $T_{c}$ of 10,466 $J/m^{2}$, whereas EPDM 90A showed a relatively lower value of 2485 $J/m^{2}$.
2. Fully-Relaxing Fatigue Crack Growth (FCG): Cyclic loading was applied to a notched pure shear specimen under conditions where the stress completely unloaded to zero (R=0). By plotting the crack growth rate dc/dN against the tearing energy, the material constants for the Thomas power law regime—namely, the crack growth rate at critical value ($r_{c}$) and the initial power law slope ($F_{0}$)—were derived. EPDM 60A was evaluated as having an initial power law slope $F_{0}=2.52$ and a crack growth rate at critical value $r_{c}=0.0601$ mm/cycle. For EPDM 90A, these parameters were evaluated as $F_{0}=5.53$ and $r_{c}=4.38$ mm/cycle.
3. Non-Relaxing Fatigue Crack Growth (FCG): To simulate the continuous compression state occurring in the actual operating environment of O-rings, cyclic tests were conducted under positive load ratio (R>0) conditions for EPDM 60A. Based on the shift in the obtained crack growth curves, the material parameters for the Mars-Fatemi strain crystallization model were evaluated with an initial power law slope $F_{0}=2.52$ and a power law exponent $F_{exp}=0.739$.
4. Initial crack diameter (*c*_0_) calibration and configuration: Unnotched fatigue-test results were compared with numerical life outputs from an inverse-analysis tensile-specimen model. For EPDM 60A, numerical cases with *c*_0_ = 0.0080, 0.0096, and 0.0120 mm were compared with the test results at 100% and 200% nominal strain, and *c*_0_ = 0.0096 mm was selected as the material-specific effective precursor input. For EPDM 90A, the inverse comparison with the unnotched fatigue-test observations at 40% and 90% nominal strain yielded *c*_0_ = 0.0085 mm as the material-specific effective precursor input. The initial-crack-size inverse-calibration model contained 7725 nodes and 5712 linear hybrid hexahedral elements (C3D8RH); these mesh counts pertain to the tensile-specimen calibration model and not to the O-ring model. The available comparisons are shown in Figure 3. The *c*_0_ inputs listed in Table 5 were subsequently used in the fe-safe/Rubber life integration. These inverse-calibrated values are effective precursor inputs and do not establish physical crack-nucleation sizes in the O-ring or provide a causal explanation for the pressure-dependent ordering of the two material-specific model outputs.

## 4. Computational Modeling and Automation Framework

**Figure 4:**
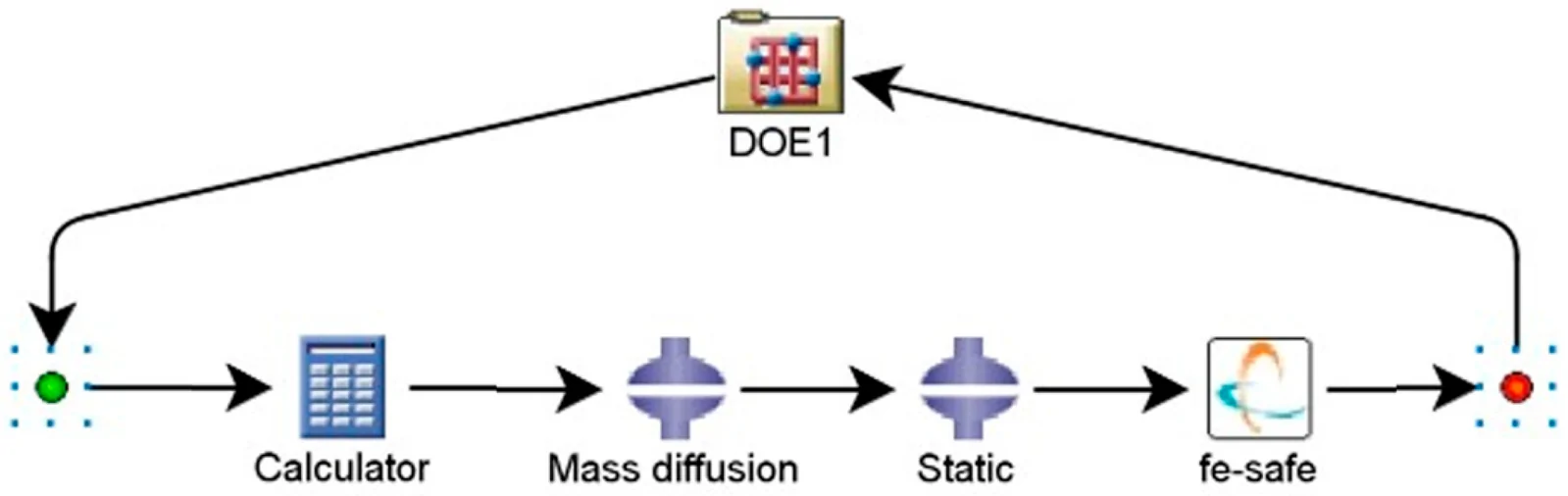
Isight-based automated sequential workflow linking Abaqus mass-diffusion and structural analyses with downstream fe-safe/Rubber fatigue post-processing.

The experimentally characterized and prescribed model inputs were organized within an Isight 2019-based automated sequential screening workflow. Abaqus/Standard 2019 was used for mass-diffusion and nonlinear structural/contact analyses, and fe-safe/Rubber 2019 was used for downstream fatigue post-processing. Isight 2019 advanced through a predefined material–pressure–stretch design matrix and routed the required inputs and outputs between analysis stages. The workflow is automated at the case-execution level, but the physical analyses are one-way and sequential rather than monolithically or bidirectionally coupled. No optimization objective or certified design limit is derived in the present study. The workflow architecture is summarized in Figure 4.

### 4.1. Mass Diffusion Modeling Configuration

Hydrogen transport in the axisymmetric O-ring was modeled with DCAX4 mass-diffusion elements. The initial modeled hydrogen concentration was set to zero. The lower O-ring surface exposed to hydrogen was assigned the normalized concentration boundary *ϕ* = 1 for a steady-state charging analysis. For decompression, the exposed-surface boundary was changed to zero and a transient mass-diffusion step of 86,400 s was performed. The material-specific diffusivities were 2.9 × 10^−10^ m^2^/s (2.9 × 10^−4^ mm^2^/s) for EPDM 60A and 2.3 × 10^−10^ m^2^/s (2.3 × 10^−4^ mm^2^/s) for EPDM 90A. The same material-specific *D*_H_ was used at 3, 7, 14, and 21 MPa; this constant-*D*_H_ pressure transfer is a modeling assumption. The pressure-dependent wt.ppm values in Table 4 were entered directly in the Abaqus solubility-input field as the empirical normalization scale *C*_scale_(*P*), without a separate unit conversion. They are distinct from the physical transport solubility *S*_H_; both *C*_H_ and *C*_scale_(*P*) were treated on the same wt.ppm basis in *ϕ* = *C*_H_/*C*_scale_(*P*). The normalized concentration output NNC from charging and decompression was transferred one way to the structural analysis as predefined fields.

### 4.2. Prescribed Swelling and Structural Analysis

**Figure 5:**
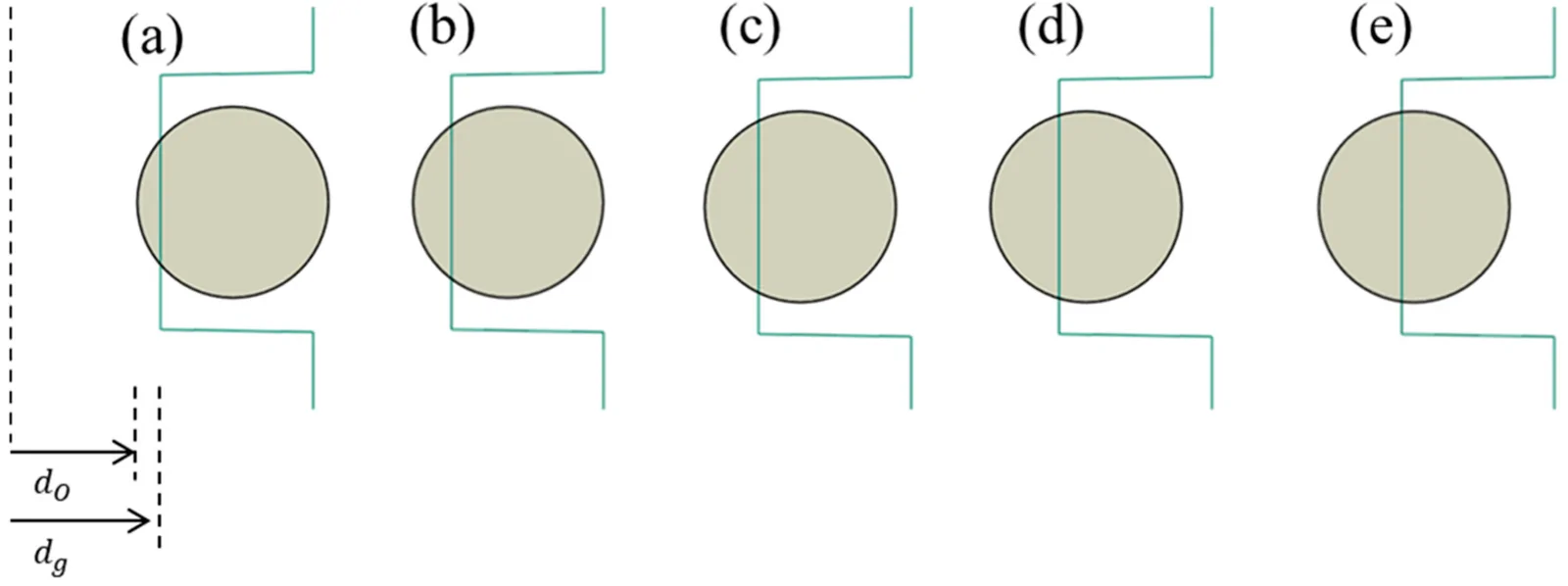
Initial axisymmetric O-ring geometries corresponding to circumferential installation stretches of 1–5%. Radial squeeze was fixed at 20% for every design point and was applied during the subsequent interference-fit assembly. Subfigures (**a**–**e**) correspond to initial stretches of 1%, 2%, 3%, 4%, and 5%, respectively. The green lines denote the groove boundaries, and the circles denote the O-ring cross-sections.

The computational O-ring model used a nominal two-dimensional axisymmetric configuration. The O-ring cross-section diameter was 6.99 mm; the 1% installation-stretch base model had a centerline diameter of 122.119 mm. The groove width and radial depth were 9.5 and 5.59 mm, respectively, the pipe inner diameter was 128 mm, and the housing/groove inner diameter was 116.82 mm. The groove corner was sharp, no extrusion clearance gap was included, and the pipe and groove were represented as analytical rigid surfaces. The deformable O-ring mesh contained 308 elements and 337 nodes with a nominal characteristic element size of 0.388 mm. DCAX4 and CAX4RH elements were used for diffusion and structural analyses, respectively, with identical mesh topology. Contact between the O-ring and the pipe/groove surfaces used hard normal contact, penalty tangential behavior with a friction coefficient of 0.1, finite sliding, and separation after contact. All Abaqus inputs were expressed in a consistent mm–tonne–s unit system; *D*_H_ was therefore entered in mm^2^/s, and pressure was expressed in MPa. The axisymmetric formulation assumes circumferential uniformity and does not resolve circumferentially localized three-dimensional asymmetry; the reported cases therefore represent nominal screening configurations. The NNC concentration histories obtained from the diffusion analysis were transferred to the structural model as predefined fields. The structural solution did not feed back to the diffusion calculation. The structural simulation proceeded sequentially as follows:

1. Initial Pre-stretch Configuration: Circumferential installation stretch was varied from 1% to 5% by adjusting the initial O-ring geometry before groove assembly. The 1% base configuration had a centerline diameter of 122.119 mm. Radial squeeze was fixed at 20% for all design points and was not varied as a DOE factor. The initial geometries are shown in Figure 5.
2. Groove Assembly and Radial Compression: The pipe/groove geometry was positioned to provide a nominal 20% radial squeeze relative to the 6.99 mm O-ring cross-section, and the assembly deformation was established using the Abaqus interference-fit procedure. The 20% squeeze was held fixed throughout the reported pressure–stretch matrix.
3. Fluid Pressurization and Prescribed Swelling: Mechanical fluid pressure was applied to the same lower O-ring surface used as the hydrogen-exposure boundary. The pressure-dependent prescribed swelling input *α*_sw,eff_(*P*) was scaled by the transferred normalized concentration field according to the swelling relation defined in Section 2.3 and applied isotropically in the structural analysis. This constitutes a one-way concentration-to-structure transfer; no structural feedback to the diffusion solution was implemented.
4. Cyclic Structural Loading: After interference-fit assembly, the structural analysis applied pressure cycle 1, decompression cycle 1, pressure cycle 2, and decompression cycle 2. Exactly two pressure–decompression cycles were analyzed, and the Cycle-2 structural history was transferred to fe-safe/Rubber. No quantitative hysteresis-stabilization or cyclic-convergence criterion was established; Cycle 2 is therefore not described as a stabilized or converged equilibrium cycle.

### 4.3. Tearing Energy-Based Fatigue Analysis via Fe-Safe/Rubber

The Cycle-2 Abaqus structural history was processed downstream in fe-safe/Rubber using the material-specific fatigue definitions summarized in Table 5. The fatigue calculation did not modify the Abaqus structural solution, contact state, geometry, or diffusion field. Isight collected Life-Repeats. Value as the reported fatigue output for each predefined design point. The fatigue stage represents tearing-energy-based small-crack life integration and does not explicitly simulate crack nucleation, an evolving crack path, or structural rupture.

### 4.4. Isight Automation Architecture

**Figure 6:**
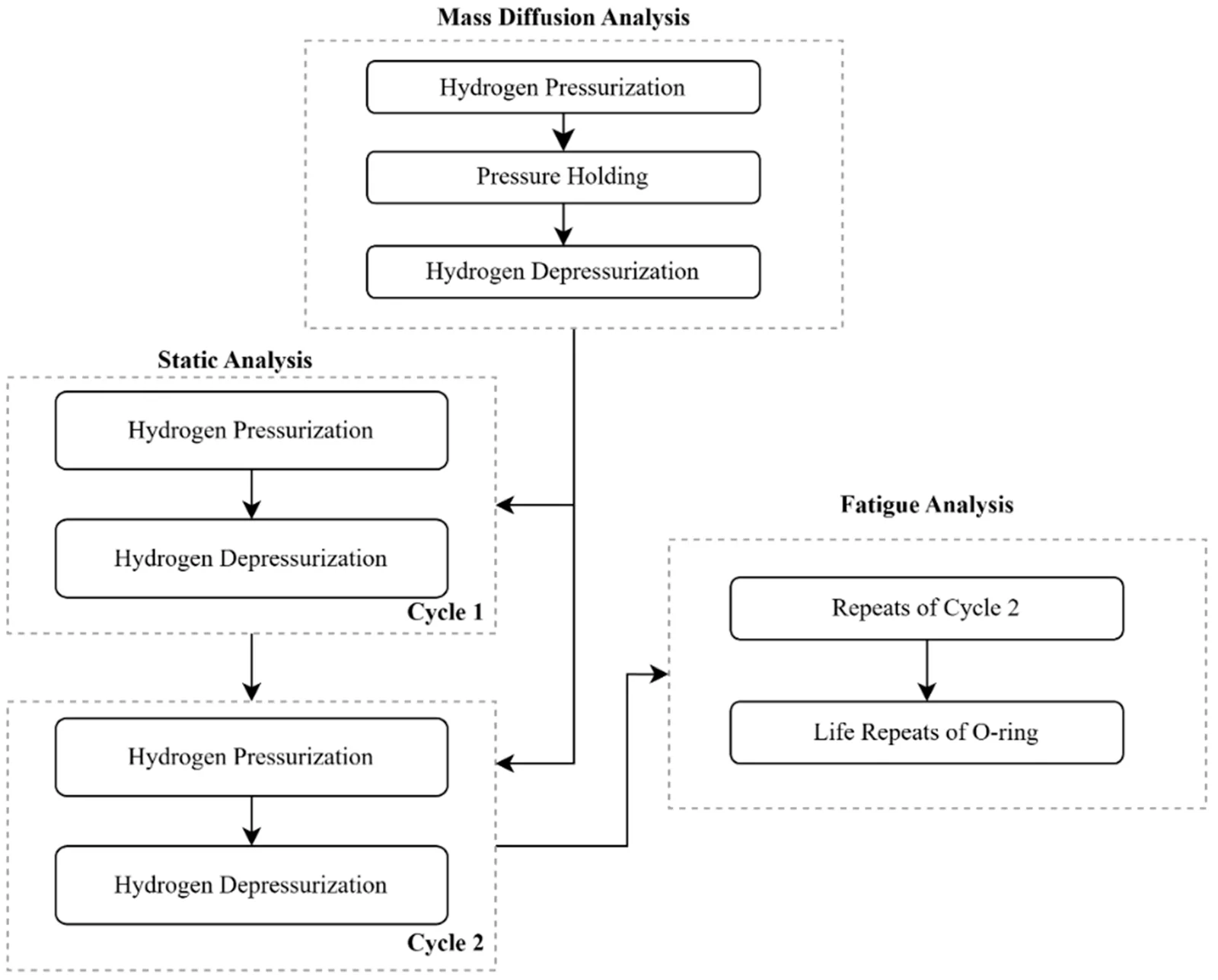
Schematic sequential structural loading and downstream fatigue-transfer sequence. Two pressure–decompression cycles were analyzed, and the Cycle-2 structural history was transferred to fe-safe/Rubber; no stabilization criterion was imposed.

Isight automated execution of a predefined deterministic design matrix comprising two material definitions, four pressure points (3, 7, 14, and 21 MPa), and five circumferential installation stretches (1–5%), while radial squeeze remained fixed at 20%. The workflow routed the material- and pressure-dependent inputs, executed the diffusion and structural analyses, passed the Cycle-2 structural history to fe-safe/Rubber, and collected the selected structural and fatigue outputs. The four reported pressure points are discrete design points; the workflow does not establish a continuous pressure response, threshold, allowable pressure, or optimization result. Figure 6 summarizes the sequential loading and transfer sequence.

**Figure 7:**
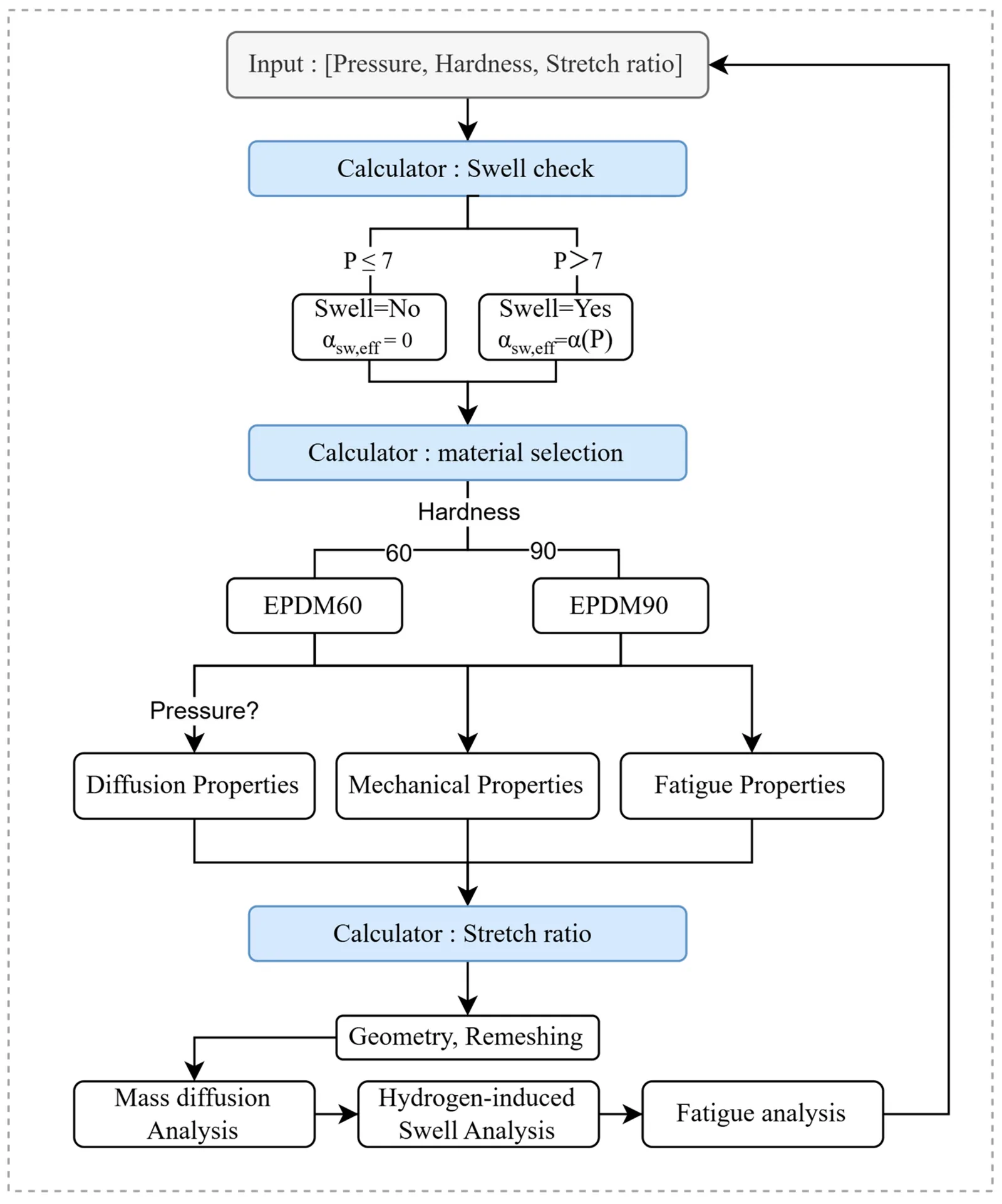
Isight deterministic parametric-execution diagram. The *P* ≤ 7/*P* > 7 and Swell = No/Yes labels denote numerical routing of the prescribed swelling inputs and do not define a physical swelling threshold. The return loop advances to the next predefined DOE case and does not represent feedback between the physical analyses. Calculator boxes denote Isight Calculator components, whereas the remaining colors distinguish other functional component types. The Input component supplies the values determined by the DOE.

The Isight routing logic used the selected material definition and pressure to assign the empirical hydrogen-content scale and prescribed swelling input. At the reported 3 and 7 MPa design points, *α*_sw,eff_ was set to zero. At 14 and 21 MPa, the material-specific values derived from the measured post-decompression volume changes listed in Table 3 were assigned. Piecewise-linear expressions were retained in the Isight project between 7 and 14 MPa and between 14 and 21 MPa, but the results reported here were generated only at 3, 7, 14, and 21 MPa; no intermediate-pressure or outside-range result is reported. Accordingly, the *P* ≤ 7/*P* > 7 and Swell = No/Yes branches shown in Figure 7 denote numerical input routing and not a physical swelling threshold. In the diagram, Hardness denotes selection of the EPDM 60A or 90A material-specific input set rather than a continuous hardness law.

The workflow collected the spatial maximum NE Mises over the deformable O-ring from the pressure step as the reported structural metric and Life-Repeats. Value as the reported fatigue output for each predefined design point. Numerical CPRESS results are not used in the revised manuscript because the archived extraction definition was insufficient to support a reproducible interface-wide sealing metric or leakage conclusion. Figure 7 summarizes the deterministic parameter-routing and execution logic. The return arrow advances Isight to the next predefined DOE case and does not represent fatigue-to-input or other physical feedback between the analyses.

## 5. Results and Discussion

### 5.1. Scope of the Prescribed Swelling Implementation

The pressure-dependent swelling inputs were derived from post-decompression free-specimen volume-change measurements and applied in the structural model using the normalized concentration field transferred from the diffusion analysis, as described in Section 2.3 and Section 4.2. Because those measurements also formed the basis of the prescribed swelling inputs and the available records did not establish the exact output-extraction procedure for the free-plate calculation, that calculation is not used as independent validation. No validation of the swelling response of the groove-constrained O-ring was performed.

### 5.2. Structural Strain Outputs Under the Sampled Pressure Conditions

Table 6 summarizes the pressure-step spatial maximum NE Mises value over the deformable O-ring for the 1% initial-stretch condition. For EPDM 60A, the reported pressure-step maximum increased from 0.51 at 3 MPa to 0.63, 0.73, and 0.84 at 7, 14, and 21 MPa, respectively. The corresponding EPDM 90A values were 0.45, 0.45, 0.52, and 0.58. At 3 and 7 MPa, the prescribed swelling input was set to zero, so the transferred concentration field had no structural pathway through the swelling term under those modeling conditions, although the mechanical fluid pressure remained active. At 14 and 21 MPa, the nonzero prescribed swelling input contributed to the calculated structural history. These values are deterministic outputs for the nominal axisymmetric configuration and do not isolate swelling as a unique cause of the pressure-dependent response.

At the 1% initial-stretch condition, the pressure-step maximum NE Mises increased across all four sampled pressures for EPDM 60A, whereas the EPDM 90A value was unchanged from 3 to 7 MPa and then increased at 14 and 21 MPa. EPDM 60A gave the larger numerical value at each sampled pressure. The present analysis does not evaluate leakage or establish a sealing margin; CPRESS outputs are not reported or interpreted. In addition, no extrusion clearance gap was included in the modeled configuration, so localized extrusion failure was not evaluated. The structural-output differences between the two compounds are reported descriptively and are not attributed to unmeasured differences in crosslink density, filler loading, or formulation.

### 5.3. Pressure-Dependent Model-Predicted Fatigue-Life Outputs

Downstream fe-safe/Rubber post-processing of the Cycle-2 structural histories produced a deterministic model-predicted Life-Repeats. Value output for each prescribed design point.

**Figure 8:**
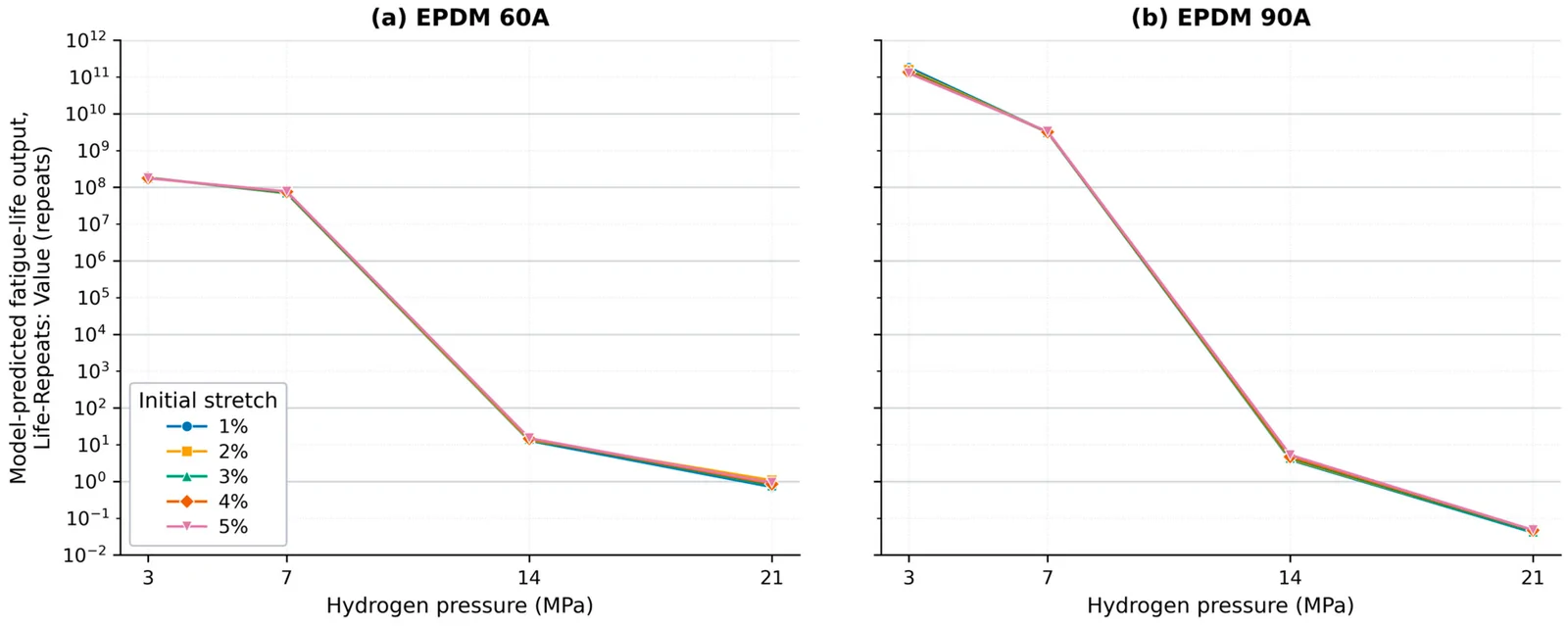
Model-predicted fatigue-life outputs at the sampled pressure and initial-stretch design points for (**a**) EPDM 60A and (**b**) EPDM 90A. The ordinate uses a base-10 logarithmic scale. Markers denote the simulated pressure points, and connecting lines, where shown, are visual guides only and do not define a continuous pressure response beyond the sampled points.

Table 7 summarizes the 1% initial-stretch results, whereas Section 5.4 reports the complete 1–5% matrix. Figure 8 displays the same discrete design-point outputs on a logarithmic ordinate. These quantities are reported only as model-predicted fatigue-life outputs; no direct component-level or within-cycle physical interpretation is assigned to them.

At 1% initial stretch, the EPDM 60A model produced outputs of 1.829 × 10^8^, 6.877 × 10^7^, 12.923, and 0.712 repeats at 3, 7, 14, and 21 MPa, respectively. The corresponding EPDM 90A outputs were 1.802 × 10^11^, 3.155 × 10^9^, 3.896, and 0.041 repeats. Both material-specific model configurations therefore exhibited a sharp numerical reduction between the sampled 7 and 14 MPa design points. Because no intermediate-pressure fatigue simulations were performed, the results do not resolve a continuous transition or any intermediate pressure boundary beyond the four sampled points.

The fractional outputs at 21 MPa are retained as genuine numerical results of the configured fatigue-life integration. They do not identify a within-cycle physical event or a specific damage mode. Case-specific *T*_max_ or *T*_eq_ outputs were not preserved; therefore, the present results do not demonstrate that the high-pressure cases exceeded *T*_c_ or establish whether they lie inside or outside the directly sampled fatigue-crack-growth range.

The ordering of the material-specific outputs changed between the sampled 7 and 14 MPa design points: the EPDM 90A configuration produced larger outputs at 3 and 7 MPa, whereas the EPDM 60A configuration produced larger outputs at 14 and 21 MPa. This sampled-point change in ordering is described as a model-output crossover; because no intermediate pressures were simulated, a crossover pressure is not identified. Because the two materials use non-equivalent fatigue-characterization bases and differ simultaneously in multiple measured, calibrated, derived, and prescribed inputs, this crossover is not interpreted as intrinsic material superiority or attributed to a single *T*_c_, hardness, swelling, crystallization, filler, or crosslink mechanism.

### 5.4. Initial-Stretch Sensitivity at Fixed 20% Squeeze

The initial circumferential installation stretch was varied from 1% to 5% while radial squeeze was held fixed at 20% for every design point. Table 8 reports the corresponding pressure-step maximum NE Mises values. Because squeeze was not varied, this analysis evaluates only initial-stretch sensitivity within the prescribed fixed-squeeze configuration and does not establish a stretch–squeeze interaction or an installation recommendation.

The pressure-step structural metric showed condition-dependent changes with installation stretch. For EPDM 60A, the 1–5% endpoint values were 0.51 and 0.51 at 3 MPa, 0.63 and 0.62 at 7 MPa, 0.73 and 0.71 at 14 MPa, and 0.84 and 0.81 at 21 MPa. For EPDM 90A, the corresponding endpoint values were 0.45 and 0.40, 0.45 and 0.41, 0.52 and 0.50, and 0.58 and 0.57. The intermediate values are reported in Table 8. These deterministic trends do not isolate hardness or any other single material characteristic as their cause. The complete model-predicted fatigue-life matrix for the 1–5% initial-stretch range is reported in Table 9.

The fatigue-output sensitivity to installation stretch was also condition-dependent. For EPDM 60A, the output decreased slightly from 1.829 × 10^8^ to 1.762 × 10^8^ repeats across 1–5% stretch at 3 MPa, increased from 6.877 × 10^7^ to 7.712 × 10^7^ at 7 MPa, and increased from 12.923 to 15.045 at 14 MPa. At 21 MPa, the EPDM 60A sequence was non-monotonic (0.712, 1.073, 0.776, 0.853, and 0.930). For EPDM 90A, the output decreased from 1.802 × 10^11^ to 1.266 × 10^11^ at 3 MPa, varied only modestly around 3.1–3.3 × 10^9^ at 7 MPa, and increased from 3.896 to 5.253 at 14 MPa and from 0.041 to 0.048 at 21 MPa. Accordingly, the present matrix does not establish a uniform beneficial effect of increasing installation stretch.

Because radial squeeze remained fixed at 20%, these results do not quantify squeeze sensitivity or stretch–squeeze interaction and do not establish a preferred installation setting.

These deterministic output trends are specific to the prescribed nominal axisymmetric model, material definitions, and fixed 20% squeeze. Their broader physical interpretation is subject to the experimental, model-form, and validation limitations summarized in Section 7.

## 6. Conclusions

This study implemented an automated sequential diffusion–structural–fatigue screening workflow for nominal axisymmetric EPDM O-ring configurations under selected high-pressure hydrogen exposure conditions. The workflow combined mass-diffusion analysis, one-way concentration-field transfer, nonlinear structural/contact analysis with a prescribed swelling input, and downstream fe-safe/Rubber fatigue post-processing. The main findings are summarized as follows:

1. Pressure-dependent numerical response: At 1% initial circumferential stretch and fixed 20% radial squeeze, both material-specific model configurations exhibited a sharp reduction in model-predicted fatigue-life output between the sampled 7 and 14 MPa design points. The 21 MPa cases produced fractional numerical outputs that are retained as genuine results of the configured fatigue-life integration; they do not identify a within-cycle physical event or a specific damage mode. Because no intermediate-pressure fatigue simulations were performed, the results do not resolve a continuous transition or any intermediate pressure boundary beyond the four sampled points.
2. Conditional material-specific output comparison: The ordering of the model-predicted fatigue-life outputs changed between the sampled 7 and 14 MPa design points: the EPDM 90A configuration produced larger outputs than EPDM 60A at 3 and 7 MPa, whereas the EPDM 60A configuration produced larger outputs at 14 and 21 MPa. This sampled-point change in ordering is described as a model-output crossover. Because no intermediate pressures were simulated, its pressure location is not identified. The comparison is conditional on the respective material-specific input sets and non-equivalent fatigue-characterization bases. It therefore does not establish an intrinsic ordering of the two compounds, and the present study does not isolate a single causal contribution from *T*_c_, hardness, swelling, strain-crystallization treatment, filler content, or crosslink structure.
3. Initial-stretch sensitivity at fixed squeeze: The calculated response to increasing circumferential installation stretch from 1% to 5% was material- and pressure-dependent. The pressure-step maximum NE Mises and model-predicted fatigue-life outputs did not show a uniformly beneficial response to increasing stretch, and the EPDM 60A fatigue-output sequence at 21 MPa was non-monotonic. Because radial squeeze was fixed at 20%, the present analysis does not quantify squeeze sensitivity or stretch–squeeze interaction and does not establish a preferred installation setting.

Overall, the workflow provides deterministic, input-conditional comparisons among predefined nominal cases and may help prioritize subsequent experiments or higher-fidelity analyses. Interpretation remains limited by the use of air-characterized fatigue parameters, constant material-specific diffusivity transferred from an approximately 1-bar characterization condition, the empirical transfer of free-specimen swelling observations to a groove-constrained O-ring, the non-equivalent fatigue definitions of EPDM 60A and 90A, and the absence of component-level validation. The outputs are therefore restricted to numerical screening within the modeled design matrix and are not suitable for direct component-level durability, operating-pressure, leakage, sealing, or material-selection decisions.

## 7. Limitations and Future Work

First, the two commercial EPDM compounds were manufactured and supplied by Pyeong Hwa Oil Seal and were identified by nominal Shore A hardnesses of 60 and 90. Detailed compound identifiers, batch/lot information, filler formulation, cure system, and crosslink density were confidential or otherwise unavailable, and equivalence of the material batches used across the different characterization campaigns was not independently verified. These uncharacterized formulation and batch variables are treated as uncontrolled compound-specific differences. The structural constitutive and Mullins parameters were calibrated from non-hydrogen-conditioned mechanical data and held fixed across the hydrogen-pressure analyses, while the fatigue parameters were characterized in air at approximately 23 °C. EPDM 60A included both fully relaxing and non-relaxing fatigue characterization, whereas EPDM 90A had compound-specific fully relaxing *R* = 0 fatigue-crack-growth data only and was analyzed using the software setting NOCRYSTALLIZATION. The two fatigue definitions are therefore methodologically non-equivalent, and the direction and magnitude of the bias associated with applying the 90A *R* = 0-based definition to the non-relaxing O-ring history are unknown. The inverse-calibrated c_0_ values are effective precursor inputs, and *c*_f_ is a configured numerical integration endpoint; neither identifies a physical crack-nucleation size or rupture size. In addition, the preserved high-pressure cases do not include case-specific *T*_max_ or *T*_eq_ outputs, so the applicability of the calibrated fatigue law at the highest calculated crack-driving states cannot be established from the retained records. Fractional fatigue-life outputs are retained as numerical screening indicators without assigning a within-cycle physical event or specific damage mode.

Second, the transport and swelling stages contain several input-transfer assumptions. One material-specific hydrogen diffusivity obtained under an approximately 1-bar characterization condition was held constant across the 3, 7, 14, and 21 MPa simulations; no project-specific multi-pressure *D*_H_(*P*) measurement or diffusivity-sensitivity study was available. The model used one effective concentration field and did not resolve dissolved, adsorbed, trapped, or cavity-associated hydrogen as separate species. The pressure-dependent swelling input was derived from post-decompression free-specimen volume-change measurements and transferred empirically to the constrained O-ring without a constraint-dependent correction. At 3 and 7 MPa, the prescribed swelling input was set to zero as a modeling simplification despite the small measured volume changes. The four rows in Table 4 represent four pressure points and not four replicate measurements. The original test records did not specify the statistical interpretation of the Table 2 ±values or the numbers of replicates for Table 2, Table 3 and Table 4; the records for Table 3 and Table 4 also did not indicate whether the values represented individual measurements or averages. Accordingly, these data were not treated as uncertainty bounds. No *α*_sw,eff_-sensitivity study or constrained-swelling validation was performed.

Third, the nominal two-dimensional axisymmetric model assumes circumferentially uniform geometry, material response, contact, pressure, and hydrogen exposure. It does not represent eccentric installation, manufacturing tolerances, local surface defects, asymmetric groove features, roughness, or localized three-dimensional hotspots. No extrusion clearance gap was included, so extrusion through a gap and localized extrusion failure were not evaluated. Radial squeeze was fixed at 20%, and only the 1–5% initial circumferential-stretch range was varied. Formal mesh-convergence and time-step-convergence studies were not performed. The structural analysis used two pressure–decompression cycles and transferred the Cycle-2 history to fe-safe/Rubber; no quantitative hysteresis-stabilization or cyclic-convergence criterion was established. Workflow execution was demonstrated; independent numerical verification was not demonstrated.

Fourth, the present workflow does not explicitly simulate rapid-gas-decompression cavity evolution, blister formation, cavity coalescence, true crack nucleation, an evolving geometric crack path, element failure, structural rupture, or leakage. Experimental scatter and uncertainty were not propagated through the sequential workflow, and no formal uncertainty-quantification or reliability analysis was performed. No component-level cyclic high-pressure-hydrogen fatigue test or leakage test was available to validate the reported O-ring outputs. These limitations restrict the present results to deterministic, input-conditional screening of the modeled nominal cases. Further work would be required to establish component-level durability or sealing performance, including complete compound traceability; replicated transport and swelling characterization; hydrogen-conditioned constitutive and fatigue characterization with reassessment of the Mullins inputs; matched non-relaxing characterization for EPDM 90A; fatigue-crack-growth characterization spanning the relevant calculated crack-driving range with retention of case-specific *T*_max_ and *T*_eq_ outputs; multi-pressure hydrogen-transport characterization and diffusivity-sensitivity studies; constrained-swelling characterization; three-dimensional analyses; formal mesh, time-step, and cyclic-convergence studies with independent numerical verification; uncertainty propagation; and component-level fatigue and leakage testing.

## Figures and Tables

**Table 1:** Reduced-Polynomial and Mullins parameters used in the reported O-ring analyses.

Model	Parameter (Unit)	EPDM 60A	EPDM 90A
Reduced Polynomial, *N* = 3	*C*_10_ (MPa)	0.664293	3.468592772
	*C*_20_ (MPa)	−0.055174	0.136585199
	*C*_30_ (MPa)	0.021166	0.03277731417
	$D_{1}^{\mathrm{RP}}$ (MPa^−1^)	5.0 × 10^−4^	7.974822932 × 10^−4^
	$D_{2}^{\mathrm{RP}}$ (MPa^−1^)	0	1.566955566 × 10^−5^
	$D_{3}^{\mathrm{RP}}$ (MPa^−1^)	0	9.567224121 × 10^−6^
Abaqus built-in Mullins	*r* (−)	4.149348	1.69579408
	*m* (MPa)	0.194389	0.4743914644
	*β* (−)	0.077906	0.1816293598

Note: For EPDM 60A, $D_{2}^{\mathrm{RP}}$ and $D_{3}^{\mathrm{RP}}$ were zero. The nonzero higher-order compressibility inputs for EPDM 90A are retained as defined in the implemented Reduced-Polynomial material branch. The Mullins effect was implemented using the Abaqus built-in formulation; no UMULLINS routine was used.

**Table 2:** Hydrogen-transport properties measured under the approximately 1-bar test condition and diffusivities used in Abaqus.

Material	*P*_H_ [10^−9^ mol·m/ (m^2^·s·MPa)]	Source *D*_H_ [10^−10^ m^2^/s]	*D*_H_ Used in Abaqus (m^2^/s)	*D*_H_ Used in Abaqus (mm^2^/s)	*S*_H_ [mol/(m^3^·MPa)]
EPDM 60A	7.0 ± 0.9	2.9 ± 0.2	2.9 × 10^−10^	2.9 × 10^−4^	24.1 ± 1.5
EPDM 90A	6.4 ± 0.2	2.3 ± 0.0	2.3 × 10^−10^	2.3 × 10^−4^	27.5 ± 0.7

Note: The original test records did not specify whether the ‘±’ values represent standard deviations, standard errors, or measurement uncertainties, and the number of test replicates was unavailable. The absolute diffusivities listed here were used in the Abaqus analyses. The physical transport solubility *S*_H_ is distinct from the empirical high-pressure wt.ppm input scale *C*_scale_(*P*).

**Table 3:** Post-decompression free-specimen volume changes, derived equivalent isotropic strains, and prescribed structural swelling inputs.

Material	Pressure (MPa)	Measured Δ*V*/*V*_0_ (%)	*ε*_free,eq_ (−)	Prescribed *α*_sw,eff_ (−)
EPDM 60A	3	−0.20	−0.000667112	0
	7	+0.69	+0.002294730	0
	14	+7.13	+0.023223176	0.023
	21	+14.96	+0.047568068	0.048
EPDM 90A	3	−0.13	−0.000433521	0
	7	−0.21	−0.000700491	0
	14	+3.26	+0.010750675	0.0108
	21	+5.38	+0.017621010	0.0176

Note: *ε*_free,eq_ = (1 + Δ*V*/*V*_0_)^(1/3)^ − 1. The zero prescribed inputs at 3 and 7 MPa are modeling simplifications and do not replace the measured values.

**Table 4:** Measured hydrogen contents at four pressure points used directly as the empirical wt.ppm Abaqus solubility and normalization input *C*_scale_(*P*).

Pressure (MPa)	EPDM 60A (wt∙ppm)	EPDM 90A (wt∙ppm)
3	160.97	218.09
7	282.54	358.85
14	627.03	547.68
21	907.71	596.17

**Table 5:** Fatigue-characterization basis and fe-safe/Rubber material-definition parameters.

Item	EPDM 60A	EPDM 90A
Test environment	Air, approximately 23 °C	Air, approximately 23 °C
Fully relaxing FCG data	Available (*R* = 0)	Available (*R* = 0)
Non-relaxing FCG data	Available for non-relaxing *R* > 0 loading histories; no single representative *R*	Not available
FCGR type	THOMAS	THOMAS
Load-ratio/crystallization treatment	MARSFATEMI	NOCRYSTALLIZATION (software setting)
*T*_c_ (J/m^2^)	10,466	2485
*F*_0_ (−)	2.52	5.5278
*r*_c_ (mm/cycle)	0.0601	4.3777
*F*_exp_ (−)	0.739277	Not used
*c*_0_ (mm)	0.0096	0.0085
*c*_f_ (mm)	1	5

Note: The two fatigue definitions are methodologically non-equivalent. NOCRYSTALLIZATION is a solver/material-definition setting and is not experimental proof of absent strain crystallization. The *c*_0_ values were selected by the inverse comparisons described in Section 3.3 and Figure 3 and are treated as effective precursor inputs rather than measured physical crack-nucleation sizes in the O-ring. The configured *c*_f_ values are numerical integration endpoints and are not interpreted as physical rupture sizes.

**Table 6:** Pressure-step maximum NE Mises over the O-ring at 1% initial stretch and fixed 20% squeeze.

Material	Pressure (MPa)	Maximum NE Mises (−)
EPDM 60A	3	0.51
	7	0.63
	14	0.73
	21	0.84
EPDM 90A	3	0.45
	7	0.45
	14	0.52
	21	0.58

Note: Values are the spatial maxima of NE Mises extracted from the pressure step and are dimensionless. Values are rounded to two decimal places. Radial squeeze was fixed at 20%.

**Table 7:** Model-predicted fatigue-life outputs for the 1% initial-stretch condition.

Pressure (MPa)	EPDM 60A	EPDM 90A
3	1.829 × 10^8^	1.802 × 10^11^
7	6.877 × 10^7^	3.155 × 10^9^
14	12.923	3.896
21	0.712	0.041

Note: Values are deterministic fe-safe/Rubber Life-Repeats. Value outputs (repeats). Table 7 is the 1% initial-stretch subset of the complete matrix reported in Section 5.4. Fractional values are retained as numerical screening outputs and do not identify a within-cycle physical event.

**Table 8:** Pressure-step maximum NE Mises over the O-ring as a function of initial circumferential stretch at fixed 20% squeeze.

Material	Pressure (MPa)	1%	2%	3%	4%	5%
EPDM 60A	3	0.51	0.51	0.51	0.51	0.51
	7	0.63	0.63	0.63	0.62	0.62
	14	0.73	0.72	0.71	0.71	0.71
	21	0.84	0.83	0.81	0.81	0.81
EPDM 90A	3	0.45	0.43	0.42	0.41	0.40
	7	0.45	0.44	0.43	0.42	0.41
	14	0.52	0.51	0.51	0.51	0.50
	21	0.58	0.58	0.57	0.57	0.57

Note: Values are the spatial maxima of NE Mises extracted from the pressure step and are dimensionless. Values are rounded to two decimal places. Radial squeeze was fixed at 20%.

**Table 9:** Model-predicted fatigue-life output as a function of initial circumferential stretch at fixed 20% squeeze.

Material	Pressure (MPa)	1%	2%	3%	4%	5%
EPDM 60A	3	1.829 × 10^8^	1.828 × 10^8^	1.816 × 10^8^	1.782 × 10^8^	1.762 × 10^8^
	7	6.877 × 10^7^	6.987 × 10^7^	7.201 × 10^7^	7.539 × 10^7^	7.712 × 10^7^
	14	12.923	13.386	14.292	14.520	15.045
	21	0.712	1.073	0.776	0.853	0.930
EPDM 90A	3	1.802 × 10^11^	1.585 × 10^11^	1.437 × 10^11^	1.352 × 10^11^	1.266 × 10^11^
	7	3.155 × 10^9^	3.180 × 10^9^	3.138 × 10^9^	3.202 × 10^9^	3.286 × 10^9^
	14	3.896	4.097	4.396	4.659	5.253
	21	0.041	0.043	0.043	0.047	0.048

Note: Values are deterministic fe-safe/Rubber outputs in repeats. The 1% column is identical to Table 7. Fractional values are retained as numerical screening outputs and do not identify a within-cycle physical event.

## Data Availability

The data generated and analyzed in this study are available from the corresponding author upon reasonable request. Proprietary formulation and batch information was not available to the authors.
